# The ascorbate peroxidase–related protein: insights into its functioning in Chlamydomonas and Arabidopsis

**DOI:** 10.3389/fpls.2024.1487328

**Published:** 2024-10-09

**Authors:** Anna Caccamo, Fernanda Lazzarotto, Marcia Margis-Pinheiro, Joris Messens, Claire Remacle

**Affiliations:** ^1^ Genetics and Physiology of Microalgae, InBios/Phytosystems Research Unit, University of Liège, Liège, Belgium; ^2^ Redox Signaling Lab, VIB-VUB Center for Structural Biology, Vlaams Instituut voor Biotechnologie, Brussels, Belgium; ^3^ Messens Lab, Brussels Center for Redox Biology, Brussels, Belgium; ^4^ Structural Biology Brussels, Vrije Universiteit Brussel, Brussels, Belgium; ^5^ Departamento de Genética, Universidade Federal do Rio Grande do Sul, Porto Alegre, Brazil

**Keywords:** Chlamydomonas, Arabidopsis, APX-R, H_2_O_2_, phylogeny, MxxM motif, copper

## Abstract

We review the newly classified ascorbate peroxidase–related (APX-R) proteins, which do not use ascorbate as electron donor to scavenge H_2_O_2_. We summarize recent discoveries on the function and the characterization of the APX-R protein of the green unicellular alga *Chlamydomonas reinhardtii* and the land plant *Arabidopsis thaliana*. Additionally, we conduct *in silico* analyses on the conserved MxxM motif, present in most of the APX-R protein in different organisms, which is proposed to bind copper. Based on these analyses, we discuss the similarities between the APX-R and the class III peroxidases.

## Introduction

**Figure 1:**
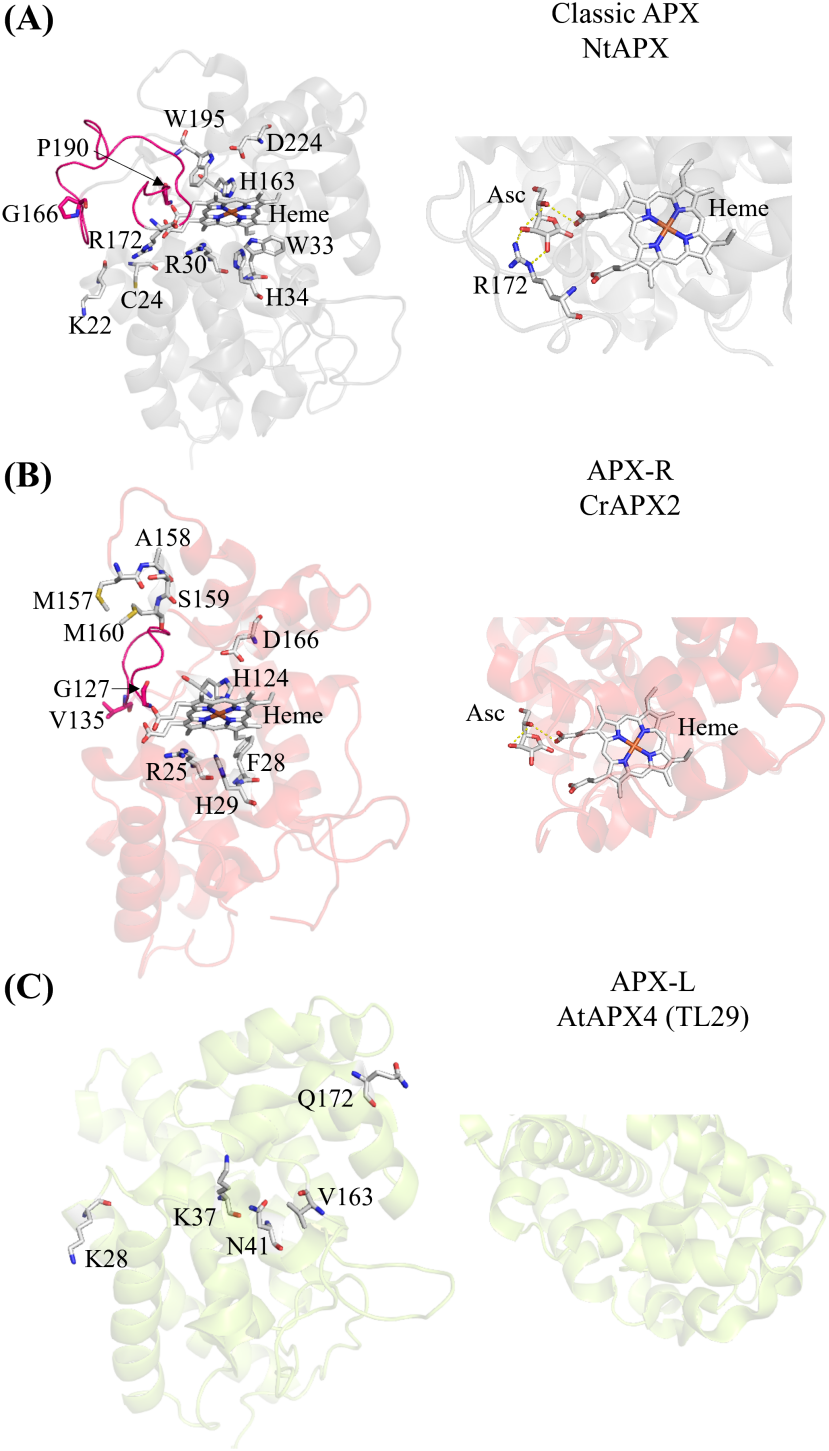
The three APX classes show structural differences. **(A)** Crystal structure of the chloroplastic APX from *Nicotiana tabacum* (Protein Data Bank (PDB) ID: 1IYN). The loop connecting two alpha helices present in the classic APX, spanning residues G166 to P190, is highlighted in pink. Key conserved amino acid residues for heme binding (H163), ascorbate binding (R172 with the contribution from K22 and C24), and catalytic activity (R30, W33, H34, H163, W195, and D224) are indicated (Lazzarotto et al., 2021a; Raven, 2003). The zoomed-in view of the predicted interaction between ascorbate (Asc), heme, and arginine (R) in classic NtAPX. **(B)** Predicted structure of the APX-R (APX2) from Chlamydomonas using AlphaFold2. The loop between G127 and V135 connecting two alpha helices highlighted in pink is shorter than the one of the classic APX **(A)**. The MxxM (MASM in Chlamydomonas) is indicated. Conserved amino acid residues for heme binding (H124) and catalytic activity (R25, F28, H29, H124, and D166) are indicated (Lazzarotto et al., 2021a; Raven, 2003). The zoomed-in view shows the predicted interaction between ascorbate (Asc) and heme. Note the absence of arginine in the APX-R of Chlamydomonas. **(C)** Crystal structure of the APX4 (TL29; APX-L) of Arabidopsis (PDB ID: 3RRW). The conserved amino acids differ from the classic APX are indicated. **(A)** heme binding (V163), the catalytic activity (K28, K37, N41) and the ascorbate binding (Q172) (Granlund et al., 2009). The zoomed-in view of APX-L (AtAPX4) reveals that there are no amino acid residues involved in binding ascorbate or heme. The prediction of ascorbate binding with arginine was aligned with the crystal structure of APX and ascorbate of *Glycine max* (PDB ID: 1OAF) using PyMol 2.5.2 (Janson et al., 2017).

Ascorbate peroxidases (APXs) are heme-containing peroxidases classified as class I peroxidases. They detoxify H_2_O_2_ by oxidizing ascorbate into monodehydroascorbate and are exclusive to photosynthetic organisms. Although their roles have been known for years (Asada, 1992; de Montellano, 2010; Foyer and Noctor, 2011; Mittler and Poulos, 2005; Shigeoka et al., 2002), recent studies have reclassified APX enzymes on the basis of their amino acid sequences (Granlund et al., 2009; Lazzarotto et al., 2021a). APX enzymes are categorized into three groups: classic APX (**Figure 1A**); APX-related (APX-R) (**Figure 1B**), which lack only the amino acids necessary for ascorbate binding; and APX-like (APX-L), which lack all the amino acids required for ascorbate and heme binding, as well as those for peroxidase activity (**Figure 1C**) (Lazzarotto et al., 2021a). Crystal structures of the classic APX [examples: (Patterson and Poulos, 1995; Wada et al., 2003; Zhang et al., 2023a)] and APX-L (Lundberg et al., 2011) are available, whereas only structural predictions exist for the APX-R (Caccamo et al., 2023). The locations of critical amino acids are indicated in the protein structures of the three APX classes in **Figure 1**.

Classical APX, found in chloroplasts, mitochondria, peroxisomes and the cytosol, function as antioxidant enzymes and redox regulators, and the cytosol function as antioxidant enzyme and redox regulator. These enzymes will not be discussed here [see recent reviews by Foyer and Hanke (2022) and Yoshimura and Ishikawa (2024)].

For APX-L, data are available for both *Arabidopsis thaliana* (hereafter Arabidopsis) and the green alga *Chlamydomonas reinhardtii* (hereafter Chlamydomonas). In Arabidopsis, APX-L (AtAPX4) is associated with photosystem II and likely involved in photosystem II photoprotection (Granlund et al., 2009), whereas, in Chlamydomonas, APX-L (CrAPX4) is crucial for cell protection under very high light conditions (Kuo et al., 2020). Granlund and colleagues (Granlund et al., 2009) found that recombinant AtAPX4 lacks ascorbate activity and does not bind heme. Additionally, peroxidase activity was unchanged between the wild-type and the AtAPX4 knockout line, suggesting that the protein does not play a stress response role. However, other studies on *apx4* mutants of both Arabidopsis (Wang et al., 2014) and Chlamydomonas (Kuo et al., 2020) reported higher H_2_O_2_ levels, suggesting a H_2_O_2_-scavenging role (Li, 2023).

For APX-R, the function has been recently described for both Chlamydomonas and Arabidopsis.

## APX-R in Chlamydomonas

In Chlamydomonas, APX2 is the APX-R protein that has been studied both *in vitro* and *in vivo*. Caccamo and co-workers (Caccamo et al., 2023) found that recombinant APX2 can reduce H_2_O_2_ without ascorbate, although the *in vivo* electron donor remains unknown. By analyzing the predicted structure of APX2 and comparing it with APX-R proteins from other organisms, they identified a conserved MxxM or MxxH motif (**Figure 1B**) typically associated with metal binding, such as copper or silver (Rubino et al., 2010). APX2 expressed in *E. coli* was shown to bind copper. The authors also noted that the heme-covering loop in classic APX differs in size (**Figure 1A**), suggesting potential protein–protein interactions.

^1^H-Nuclear Magnetic Resonance (NMR) analyses were performed to explore possible interactions between recombinant APX2 and plastocyanin, a copper-containing protein involved in electron transfer during photosynthesis. Plastocyanin, located at the luminal side of the chloroplast, is likely to interact with APX2, which has a twin-arginine-translocator motif specific for the lumen (Caccamo et al., 2023). Although no direct interaction was observed *in vitro*, the findings suggest that APX2 might affect copper insertion into plastocyanin. Indeed, Chlamydomonas mutants lacking the APX2 protein display lower plastocyanin levels compared to the wild type, impacting electron transport and the redox state of photosystem I during photosynthesis (Caccamo et al., 2024). This difference is absent under copper-deficient conditions, indicating that Chlamydomonas *apx2* mutants can switch to using the iron-containing protein cytochrome c_6_ as an alternative electron carrier. This result supports the link between the APX2 protein and plastocyanin.

**Figure 2:**
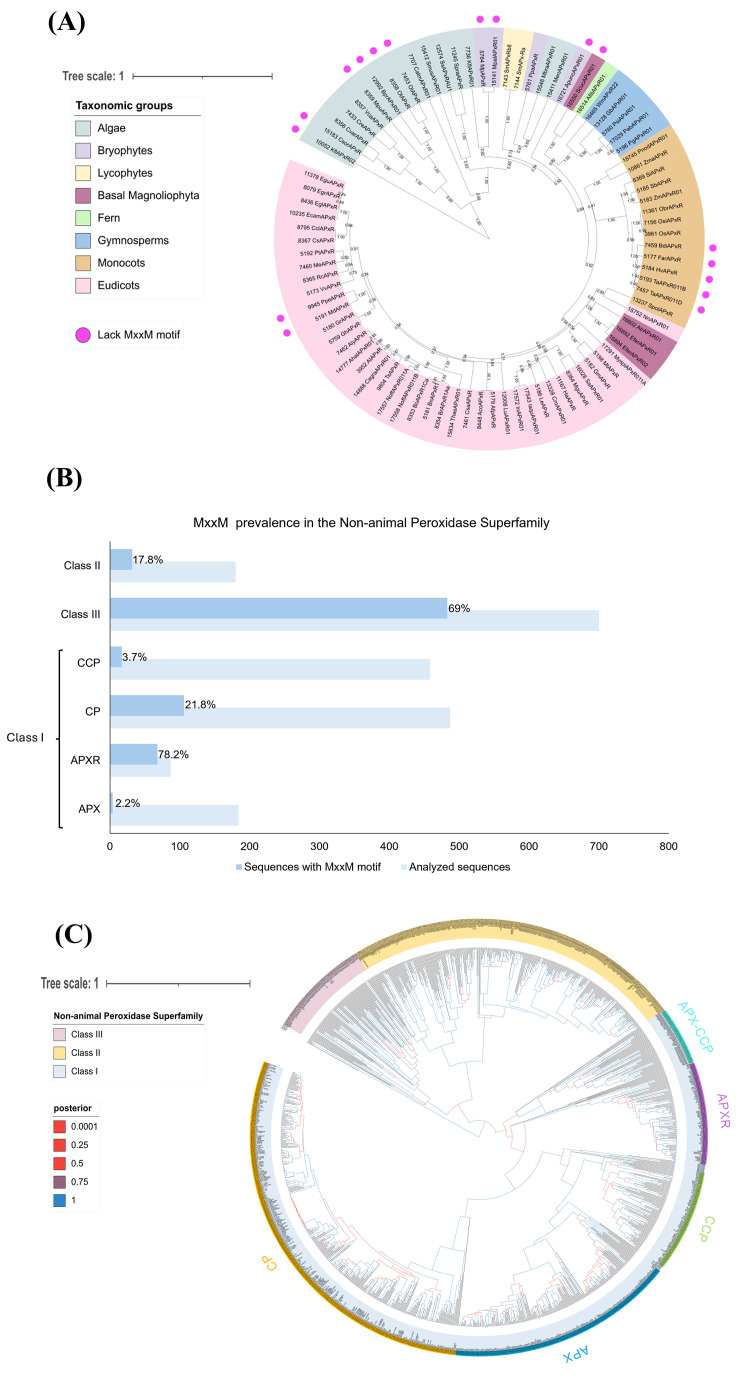
The MxxM motif is predominantly found in APX-R and class III peroxidases. **(A)** The analysis shows the phylogenetic relationship of 86 APX-R sequences, reconstructed through Bayesian inference. Out of these, 17 lack the MxxM/H motif, as indicated by pink dots. The absence of this motif occurs across sequences belonging to Algae, bryophytes, basal Magnoliophyta, monocots, and eudicots, indicating that this feature is not related to a specific taxonomic group. Posterior probabilities are indicated. **(B)** The graph shows that the MxxM motif is predominantly present in the class I APX-R and class III peroxidases. A total of 2,096 protein sequences belonging to the superfamily were examined for the presence of MxxM. In the figure, the light blue bars represent the total number of sequences analyzed for each family, whereas the darker blue bars indicate the sequences containing the MxxM motif. The percentage of sequences with the motif is displayed within the bars for each family. Protein sequences in FASTA format were analyzed for the degenerate motif MxxM using R with the Biostrings package. Sequence headers were parsed to extract labels via regular expressions, enabling grouping by predefined categories. The gregexpr function identified and quantified motif occurrences across sequences, which were then aggregated by label. The results, including total counts and percentage of sequences containing the motif, were exported as CSV files. CCP, cytochrome c peroxidase; CP, catalase peroxidase; APX-R, ascorbate peroxidase–related; and APX, ascorbate peroxidase. **(C)** The phylogenetic relationship between class I, class II, and class III peroxidases of the non-animal superfamily was reconstructed using the Bayesian method. A total of 1,102 protein sequences were included in the analysis, and ambiguous positions were removed from the alignment, with exception to amino acids implicated in the loop and in the MxxM domains. Classes I, II, and III are highlighted in blue, yellow, and pink, respectively. Class I families (CP, CCP, APX, and APX-CCP) are indicated. The posterior probabilities are shown according to the following color scheme: blue branches indicate posterior probabilities of 0.9 to 1.0; purple branches indicate posterior probabilities of 0.75 to 0.89; red branches indicate posterior probabilities inferior to 0.6. The phylogenetic analyses presented in **(A, C)** were reconstructed using conserved domains of protein sequences retrieved from RedOxiBase (Savelli et al., 2019) by Bayesian inference using BEAST (Vaughan et al., 2014). After manual inspection of the alignments, 86 sequences and 228 sites were used in the analysis **(A)** and 1,102 sequences and 336 sites in **(C)**. The best fit model of amino acid replacement was Le-Gascuel (LG) with invariable sites and gamma-distributed rates, which was selected after analyses performed on ProtTest (Abascal et al., 2005). The birth and death model was selected as tree prior, and 50,000,000 generations were performed with Markov chain Monte Carlo algorithm (MCMC) (Gilks, 2005) to evaluate posterior distributions. Convergence was verified with Tracer (Rambaut et al., 2018), and the consensus trees were generated using TreeAnnotator, part of the BEAST package. The resulting trees were analyzed and edited using FigTree v.1.4.3 (http://tree.bio.ed.ac.uk/software/figtree) and iTOL (https://itol.embl.de/).

These studies lay the foundation for understanding APX-R protein structure and function, but further research is needed to confirm the predicted structure and elucidate APX-R’s role, especially concerning the MxxM motif. Therefore, we conducted *in silico* analyses based on the APX-R sequences of Chlamydomonas and Arabidopsis to examine the presence of the MxxM motif among the green photosynthetic eukaryotes (**Figure 2**). Interestingly, the MxxM motif is widely present among the APX-R proteins in algae and plants, except for some sequences (**Figure 2A**). Some algal sequences, such as those of *Chlorella sorokiniana* (CsorAPxR), *Bathycoccus prasinos* (BprAPxR01), *Ostreococcus tauri* (OtAPxR), and *Ostreococcus lucimarinus* (OlAPxR), have a histidine instead of a second methionine (MxxH), which can also bind metals as described by Caccamo et al. (2023). Histidine is known to bind copper in various proteins, such as the pocket binding site in plastocyanin (Hill et al., 1996; Zhang et al., 2023b) or the P-type Adenosine Tri Phosphatase (ATPase) copper transporter in the thylakoid (PAA2 in Arabidopsis) (Abdel-Ghany et al., 2005).

Conversely, some sequences of plants highlighted in bryophytes, basal Magnoliophyta, monocots and eudicots, lack both methionine and histidine, suggesting that these proteins might not be involved in copper sensing. **Figure 2B** shows the prevalence of the MxxM motif in non-animal peroxidase superfamily, which includes three classes. The class I peroxidase family contains APX-Rs together with the classic APXs (found in green photosynthetic eukaryotes), catalase peroxidases (CP) (mainly found in bacteria), and the cytochrome *c* peroxidases (CCP) (found in all organisms containing mitochondria except plants). Class II includes lignin peroxidases (LiPs), manganese peroxidases (MnPs), and versatile peroxidases (VPs) found only in fungi. Class III peroxidases, found in plants, are multifunctional proteins involved in pathways such as auxin metabolism, cell wall elongation and stiffening, and pathogen protection (Mbadinga Mbadinga et al., 2020). Their phylogenetic relationship is presented in **Figure 2C**.

Notably, the MxxM motif is highly prevalent in the APX-R, present in 78.2% in 90 sequences analyzed, compared to only 2.2% in 180 classic APX sequences (**Figure 2B**). Additionally, the MxxM motif is found in class III peroxidases with 69% in 700 sequences. This analysis could provide insights into the function of APX-R, particularly in relation to the role of the MxxM motif, by comparing it to class III peroxidases.

## APX-R in land plants: focus on Arabidopsis

Because the initial identification of APX-R as a distinct peroxidase family, significant efforts have been made to clarify its role in plant antioxidant metabolism. Preliminary *in silico* analyses indicated the presence of chloroplast-targeted peptides in all examined plant APX-R proteins (Lazzarotto et al., 2011). This was later confirmed through transient expression studies in rice (*Oryza sativa*) and Arabidopsis protoplasts, as well as in transgenic Arabidopsis overexpression lines. Proteomic analyses further identified APX-R in the stroma, plastoglobuli, and thylakoid membrane, validating its subcellular localization (Lazzarotto et al., 2021b).

In Arabidopsis, the gene encoding APX-R was initially annotated as ASCORBATE PEROXIDASE6 (APX6), suggesting that it was a putative cytosolic APX gene. However, phylogenetic and biochemical evidence later demonstrated that APX-R is not part of the APX family. Although functions as a peroxidase, it does not use ascorbate as an electron donor (Lazzarotto et al., 2021a, 2021b).

Functional characterization of two knockout mutants (*apx6-1* and *apx6-3*) highlights the critical role of APX-R in oxidative protection during seed development and germination. Seeds lacking APX-R (*apx6-1*) show elevated levels of reactive oxygen species (ROS), increased oxidative damage, and reduced germination rates, particularly under stress conditions such as osmotic, salt, or heat stress. Metabolic profiling of *apx6-1* seeds revealed alterations in tricarboxylic acid cycle activity, changes in amino acid levels, and increased metabolism of abscisic acid (ABA) and auxin (Chen et al., 2014).

Studies on Arabidopsis overexpressing lines have shown that APX-R-YFP accumulates in seeds and during early development stages, underscoring its crucial role in seed metabolism and germination. APX-R is believed to protect seeds from oxidative damage during desiccation and early germination by modulating ROS and hormone signaling (Chen et al., 2014). As the plant transitions from skotomorphogenic to photomorphogenic development, APX-R-YFP undergoes degradation in most plant tissues. However, APX-R-YFP has also been observed in plant roots and stomata, suggesting that its stability may be influenced by plastid type, tissue specificity, and developmental stage (Lazzarotto et al., 2021b).

The presence of APX-R in plastoglobuli-derived samples suggests its involvement in processes related to these cellular structures, which are linked to plastid transitions during de-etiolation, senescence, and plant responses to abiotic stresses (van Wijk and Kessler, 2017). Recent studies have associated APX-R with plant senescence, showing that the Arabidopsis APX-R gene is induced in aging leaves and in response to senescence-promoting stimuli such as ABA, extended darkness, and osmotic stress. Knockout mutants exhibited early developmental senescence and increased sensitivity to dark stress (Chen et al., 2021).

Additionally, Arabidopsis *APX-R* mRNA has been identified as a potential target of miR398, a key regulator of plant copper distribution. The expression of miR398 is induced during copper deficiency and is controlled by the SQUAMOSA PROMOTER BINDING PROTEIN-LIKE7 (SPL7) transcription factor, which binds to the GTAC motif found in the miR398 promoter (Yamasaki et al., 2009). In AtSPL7 mutants, APX-R levels are higher compared to those of the wild-type plants, and this difference is further increased under copper deficiency conditions (Chen et al., 2021). These data strengthen the link between APX-R and copper, which could be relevant for understanding the role of copper in APX-R catalytic activity.

## Discussion

In this mini-review, we summarize the latest findings on the newly classified APX-R protein in algae and plants. Our comparison highlights a key common feature: the presence of the MxxM motif and its association with copper. In Chlamydomonas, this relation is linked to the electron carrier plastocyanin (Caccamo et al., 2024), a major copper storage protein in the green alga. In Arabidopsis, the APX-R-copper relation has been proposed to be dependent on copper concentrations (Chen et al., 2021), with both APX-R and copper playing roles in the senescence process (Chen et al., 2021; Hao et al., 2022). In Arabidopsis, copper is important during senescence, activating the plantacyanin-senescence associated gene (PCY-SAG14) module, with copper being redistributed from plastocyanin in the chloroplast to the cell membrane (Hao et al., 2022).

Additionally, the predicted structure of APX-R (**Figure 1**) shows another difference from classic APX: a distinctive smaller loop facing the heme group. This could also imply a possible link to metal sensitivity. Interestingly, the MxxM motif is also present in the class III peroxidases (**Figure 2**), suggesting that these proteins might bind metals as well. Class III peroxidases, found in Streptophyta, are involved in H_2_O_2_ scavenging and various processes such as germination, senescence, lignification, cell elongation (Shigeto and Tsutsumi, 2016). Predominantly, class III peroxidases can use lignin precursors, and auxin as electron donors (Hiraga et al., 2001). Our studies on the APX-R in Chlamydomonas (Caccamo et al., 2023) and Arabidopsis (Lazzarotto et al., 2021b) demonstrated that *in vitro* this protein prefers phenolic compounds like guaiacol and pyrogallol over ascorbate. Moreover, the APX-R of Arabidopsis has been showed to participate in seed germination and senescence, suggesting similarities between these two classes of peroxidases (Chen et al., 2021, 2014; Lazzarotto et al., 2021b).

There is evidence that class III peroxidases in plants respond to elevated copper levels by promoting lignin biosynthesis, which help to protect from metal stress (Ali et al., 2006; Chen et al., 2002; Lequeux et al., 2010; Lin et al., 2005; Tugbaeva et al., 2022). However, the presence and role of the MxxM motif in class III peroxidases remains unexplored, making it an exciting topic for further exploration. This raises questions whether APX-R protects cells through direct H_2_O_2_ scavenging, copper-binding, or a combination of both. Additionally, understanding why some sequences lack the MxxM (or MxxH) motif could help elucidate additional roles of APX-R (**Figure 2A**).

Additionally, information on APX-R gene expression in plants under various stress conditions provides valuable insights into its role (Chen et al., 2021, 2014; Lazzarotto et al., 2021a, 2011; Tyagi et al., 2020; Verma et al., 2022). In rice, APX-R expression is upregulated in response to aluminum stress, water deficiency, and 24 h-cold stress. In *Brassica rapa* and *B. juncea*, APX-R responds to heat and drought stresses. In *Triticum aestivum*, transcriptomic profiles of six APX-R genes show involvement in developmental stages and various biotic and abiotic stresses (e.g., fungal infection, salt, heat, and drought stresses). Moreover, Tyagi and co-authors observed that APX-R mRNA could be miRNA target (Tyagi et al., 2020). Similar regulation has been already suggested and discussed for Arabidopsis. In crops, targeting APX-R activation could be of interest. However, APX-R is a single-copy gene (Dunand et al., 2011), and overexpression in Arabidopsis leads to APX6 degradation (Lazzarotto et al., 2021b), whereas overexpressing in rice showed no alterations (Lazzarotto et al., 2011).

Further investigations are needed to explore the diversity of APX-R functions in plants and algae, the significance of the MxxM motif, and its role in copper binding. The analyses presented here offer an intriguing starting point for future research.
